# The boosting effects of melatonin on the expression of related genes to oocyte maturation and antioxidant pathways: a polycystic ovary syndrome- mouse model

**DOI:** 10.1186/s13048-022-00946-w

**Published:** 2022-01-20

**Authors:** Fatemeh Nikmard, Elham Hosseini, Mehrdad Bakhtiyari, Mahnaz Ashrafi, Fardin Amidi, Reza Aflatoonian

**Affiliations:** 1 Anatomy Department, School of Medicine, Iran University of Medical Sciences, Tehran, Iran; 2 Department of Obstetrics and Gynecology, Mousavi Hospital, School of Medicine, Zanjan University of Medical Sciences, Zanjan, Iran; 3 Zanjan Metabolic Diseases Research Center, Zanjan University of Medical Sciences, Zanjan, Iran; 4 Cellular and Molecular Research Center, School of Medicine, Iran University of Medical Sciences, Tehran, Iran; 5 Department of Endocrinology and Female Infertility, Reproductive Biomedicine Research Center, Royan Institute for Reproductive Biomedicine, ACECR, Tehran, Iran; 6 Anatomy Department, School of Medicine, Tehran University of Medical Sciences, Tehran, Iran

**Keywords:** Melatonin, PCOS, In-vitro maturation, Antioxidant

## Abstract

**Background:**

Melatonin, as a free radical scavenger exhibiting genomic actions, regulates the antioxidant genes expression and apoptosis mechanisms. In polycystic ovary syndrome (PCOS) patients, an imbalance between free radicals and antioxidants in follicular fluid leads to oxidative stress, aberrant folliculogenesis, and intrinsic defects in PCOS oocytes. In this experimental mouse model study, oocytes of PCOS and the control groups were cultured in different melatonin concentrations (10^− 5^, 10^− 6^, and 10^− 7^ M) to investigate the expression of oocyte maturation-related genes (*Gdf9*/*Bmp15*), antioxidant-related genes (*Gpx1*/*Sod1*), apoptotic biomarkers (*Bcl2*/*Bax*) and total intracellular ROS levels.

**Results:**

*Gdf9* and *Bmp15*, *Gpx1* and *Sod1* were up-regulated in PCOS and control oocytes cultured in all melatonin concentrations compared to those cultured in IVM basal medium (*P* < 0.05). A significant decrease in the total ROS level was observed in all groups cultured in the supplemented cultures. Melatonin increased *Bcl2* and decreased *Bax* gene expression in PCOS and control oocytes compared to non-treated oocytes.

**Conclusions:**

Melatonin increased antioxidant gene expression and regulated the apoptosis pathway, effectively reducing the adverse effects of culture conditions on PCOS oocytes. Furthermore, it influenced the expression of oocyte maturation-related genes in PCOS, providing valuable support during the IVM process.

## Introduction

The quality of oocyte can affect embryo development, embryo survival and the occurrence of pregnancy. The development potential of an oocyte to form an embryo is attained during its maturation [1, 2]. Immature oocytes can also be used in certain circumstances in assisted reproductive techniques (ART), where such oocyte sources are able to mature in-vitro and enter the process of embryo formation. Poor quality of oocytes after in-vitro maturation (IVM) leads to reduced fertilization rate and embryo quality. Meanwhile, one of the most critical factors affecting the outcome of IVM is oxidative stress (OS), during which an imbalance between the production of free radicals (reactive oxygen species, ROS) and antioxidant defense systems is occurred [3, 4]. Free radicals are known as molecules containing one or more unpaired electrons in their molecular orbitals leading to their instability and reactivity that react with lipids, proteins and DNA leading to their degradation. Oxidative stress can be a precursor of conditions causing women infertility, including endometriosis and polycystic ovary syndrome (PCOS) [5]. Along with pathological roles, free radicals in the physiological levels are involved in regulating female reproductive functions such as oocytes maturation, ovarian steroidogenesis, corpus luteum function, fertilization and embryonic development [6].

The success of ART outcomes in PCOS women is low, which can be related to low oocyte quality. According to the literature, one crucial element is an imbalance between free radicals and antioxidants in follicular fluid of PCOS patients, leading to oxidative stress, aberrant folliculogenesis, and intrinsic defects in PCOS oocytes [6].

Numerous studies have been conducted by adding antioxidants to the IVM media in order to reduce the impact of OS on oocytes. Melatonin, the essential hormone secreted by pineal gland, has received much attention as a broad-spectrum antioxidant and potent free radicals’ scavenger. Interestingly, a high melatonin concentration is also detected in a typical ovarian environment, and its binding sites have been distinguished in the granulosa cells [7, 8]. Thus, the relationship between melatonin and the quality of oocytes has been taken into consideration and used in IVM media research in different species. Moreover, it is significantly decreased in follicular fluid of patients with PCOS. Both clinical and experimental studies show that melatonin improves oocyte nuclear maturation, quality of follicles, ovulation, and finally, pregnancy rate in PCOS [9, 10]. Because the risk of ovarian hyperstimulation syndrome is higher in PCOS patients, IVM has usually been offered to these patients in order to use a mild-stimulation approach and reduced such complications [11].

In addition to the nuclear maturation, identifying factors affecting molecular cytoplasmic maturation of oocytes can provide an opportunity to improve in vitro maturation of oocytes. It is possible that only cytoplasmic, not nuclear maturity, is influenced in PCOS oocytes. Thus, this study aimed to investigate the impact of melatonin on mRNA expression levels of Gdf9 and Bmp15 in PCOS oocytes and its impact on the expression of antioxidant and apoptotic-related genes.

## Results

### Confirmation parameters for DHEA-induced PCOS mouse model

**Fig. 1:**
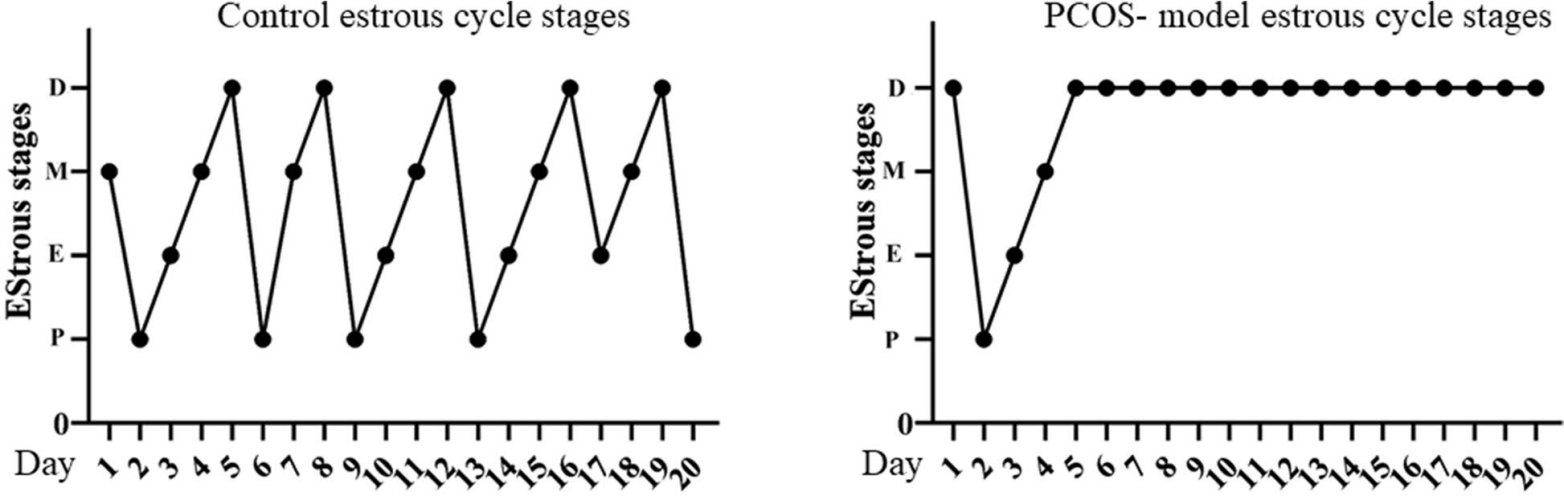
Estrous cycle changes in two representative mice from each group (Control and PCOS- model). P: proestrus; E: oestrus; M: metestrus; D: dioestrus

Confirmation parameters for creating of PCOS mouse model were also reported in the previous study [12]. Briefly, vaginal smears evaluation showed a natural cycle of 4–5 days in control mice consisting of estrus, proestrus, metestrus, and diestrus stages; whereas, constant pseudo-diestrous was observed in the PCOS model which this pattern indicated the lack of cyclic estrus in PCOS mice model (Fig. 1). Furthermore, histological analysis of ovarian tissues showed no sign of ovulation (absence of corpus luteum) in the PCOS model, and the number of antral follicles was significantly higher than the control group. Increased level of androgens is a necessary condition for induction of PCOS, which was confirmed in this study. Testosterone levels in the PCOS model significantly increased; also, there was no change in LH and FSH levels. The LH and FSH levels of the diestrus cycle were not different in both groups; whereas, there was a significant increase in the proestrus stage.

### Data analysis of melatonin’s effect on mRNA expression of *Gdf9* and *Bmp15* genes

Different analyses between groups were performed as follows:

i. between treated and untreated PCOS oocytes
ii. between treated and untreated Control oocytes
iii. between treated and untreated PCOS and control oocytes

**Fig. 2:**
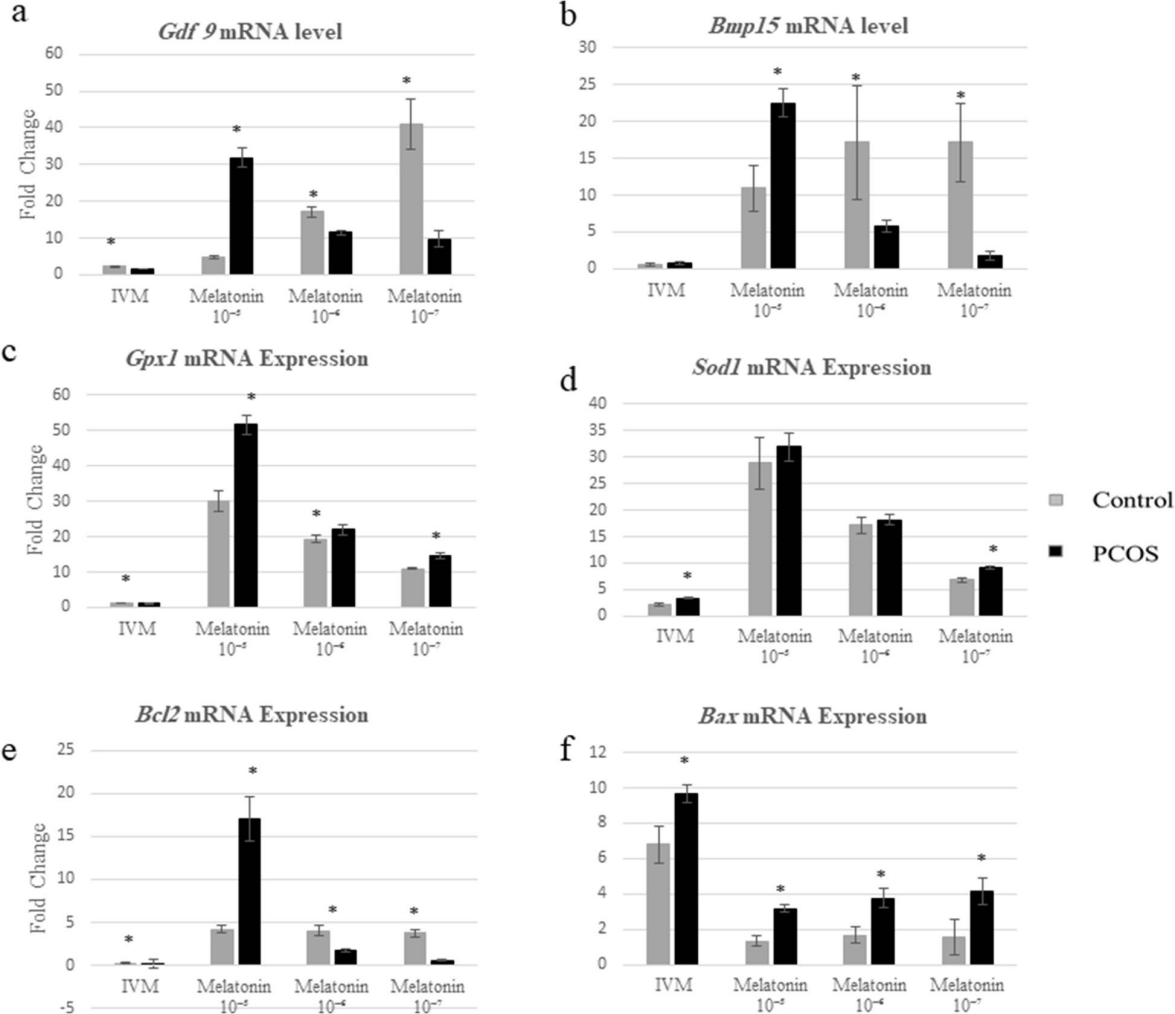
The relative expression of Gdf9, Bmp15, Gpx1, Sod1, Bcl2 and Bax genes at the mRNA level of oocytes in the melatonin treatment versus the un-treated groups as well as PCOS versus control. Bar represents by mean ± SD and the *p*-value *<* 0.05 was considered as significant

The impact of incremental melatonin doses during IVM of immature PCOS and control oocytes on the mRNA expression of *Gdf9* and *Bmp15* genes was evaluated. The effect of melatonin on *Gdf9* expression is dose-dependent. In general, *Gdf9* expression was significantly lower in the untreated PCOS oocytes than in treated ones (Fig. 2a). The maximum expression of this gene was observed in the oocytes cultured in media containing the highest concentration of melatonin (10^− 5^ M). Also, results showed that melatonin levels of 10^− 6^ and 10^− 7^ M have a positive impact on the expression of *Gdf9*; although, there was no significant difference between these two levels. The impact of these two concentrations on gene expression was significantly lower than the highest dose (Fig. 2a). When comparison was performed between PCOS and control for *Gdf9* expression, data showed that *Gdf9* expression in control untreated oocytes is higher than untreated PCOS oocytes. *Gdf9* mRNA expression in control-treated groups is significantly higher at all concentration levels, except for 10^− 5^ M, in which *Gdf9* expression is significantly higher in PCOS oocytes. Detailed results are shown in Fig. 2a.

The *Bmp15* mRNA expression in PCOS oocytes was also affected by melatonin, as it significantly increased at a concentration of 10^− 5^ and 10^− 6^ M in such oocytes; however, at low concentrations, gene expression was similar to untreated PCOS oocytes (Fig. 2b). In the control group, various concentrations of melatonin significantly increased the *Bmp15* expression compared to untreated control oocytes. Comparison of PCOS and control groups showed no difference between the two groups in terms of the *Bmp15* expression in untreated oocytes. In 10^− 5^ M PCOS treated oocytes, significantly increased gene expression was observed, while in other concentrations, the oocytes in control group abundantly expressed *Bmp15* gene. Detailed results are shown in Fig. 2b.

### Data analysis of melatonin’s effect on mRNA expression of *Gpx1* and *Sod1* expression

The usage of dose-dependent melatonin increased the expression of Gpx1 in PCOS oocytes. Therefore, the highest and lowest levels of *Gpx1* expression in treated groups were found at the 10^− 5^ and 10^− 7^ M levels of melatonin, respectively. Interestingly, *Gpx1* expression in untreated PCOS oocytes was at a lower level. On the other hand, the expression pattern of *Gpx1* in the control group was dose-dependent as well; while a comparison of the PCOS and control groups reveals that melatonin significantly increased *Gpx1* expression in PCOS oocytes at all dosages (*p* ≤ 0.05) (Fig. 2c).

*Sod1* mRNA expression in both PCOS and control groups depends on the dose of melatonin and follows the same pattern. In both groups, increasing melatonin levels resulted in a substantial increase in *Sod1* expression. In melatonin-free culture media and with a minimum concentration of melatonin (10^− 7^ M), expression of *Sod1* significantly increased in PCOS oocytes (*p* ≤ 0.05) than the control group. In addition, the *Sod1* expression increased at concentrations of 10^− 6^ and 10^− 5^, but the difference was not significant (Fig. 2d).

### Data analysis of melatonin’s effect on mRNA expression of *Bcl2* and *Bax* expression

Results showed that the *Bcl2* expression (anti-apoptotic gene) in PCOS oocytes significantly increases at high concentrations of melatonin (10^− 5^ M) (p ≤ 0.05). In contrast, the *Bcl2* expression decreased insignificantly by reducing the concentration of melatonin. The lowest *Bcl2* expression was found in melatonin-free culture media. In the control group, the *Bcl2* expression significantly increased in melatonin-treated oocytes than in melatonin-free culture media. Despite different concentrations of melatonin, the *Bcl2* expression showed no significant difference. The comparison showed that the *Bcl2* expression in the control group significantly increased in the melatonin-free media and at different concentrations of melatonin as compared to the PCOS oocytes, except that the *Bcl2* expression in PCOS oocytes was significantly higher at a concentration of 10^− 5^ M (*p* ≤ 0.05) as shown in the Fig. 2e.

Pro-apoptotic *Bax* gene expression in PCOS oocytes was enhanced in the absence of melatonin compared to melatonin-containing culture media. However, *Bax* expression is negatively associated with melatonin levels, so that the lowest expression of Bax occurred at 10^− 5^ M melatonin. Similar to PCOS results, a significant increase of *Bax* was observed in the melatonin-untreated oocytes as compared to the melatonin-treated oocytes in the control group. Different concentrations of melatonin reduced the *Bax* expression in the control oocytes, but there was no significant difference between different concentrations of melatonin in this group. It was shown that the *Bax* gene expression was considerably greater in PCOS oocytes when compared to those of healthy controls (*p* ≤ 0.05). Figure 2f shows details of the *Bax* expression.

### Data analysis of the melatonin’s effect on intracellular ROS level

In PCOS and control oocytes, the effect of melatonin concentration on the neutralization of free radicals was studied. Melatonin-untreated oocytes had the highest concentration of free radicals, according to studies of fluorescence intensity. The concentration of free radicals in the culture medium decreased as the concentration of melatonin in the culture medium increased. The lowest and highest concentrations of free radicals were observed in media containing 10^− 5^ M and in the melatonin-free media respectively. The results were identical in both the control and PCOS groups.

**Fig. 3:**
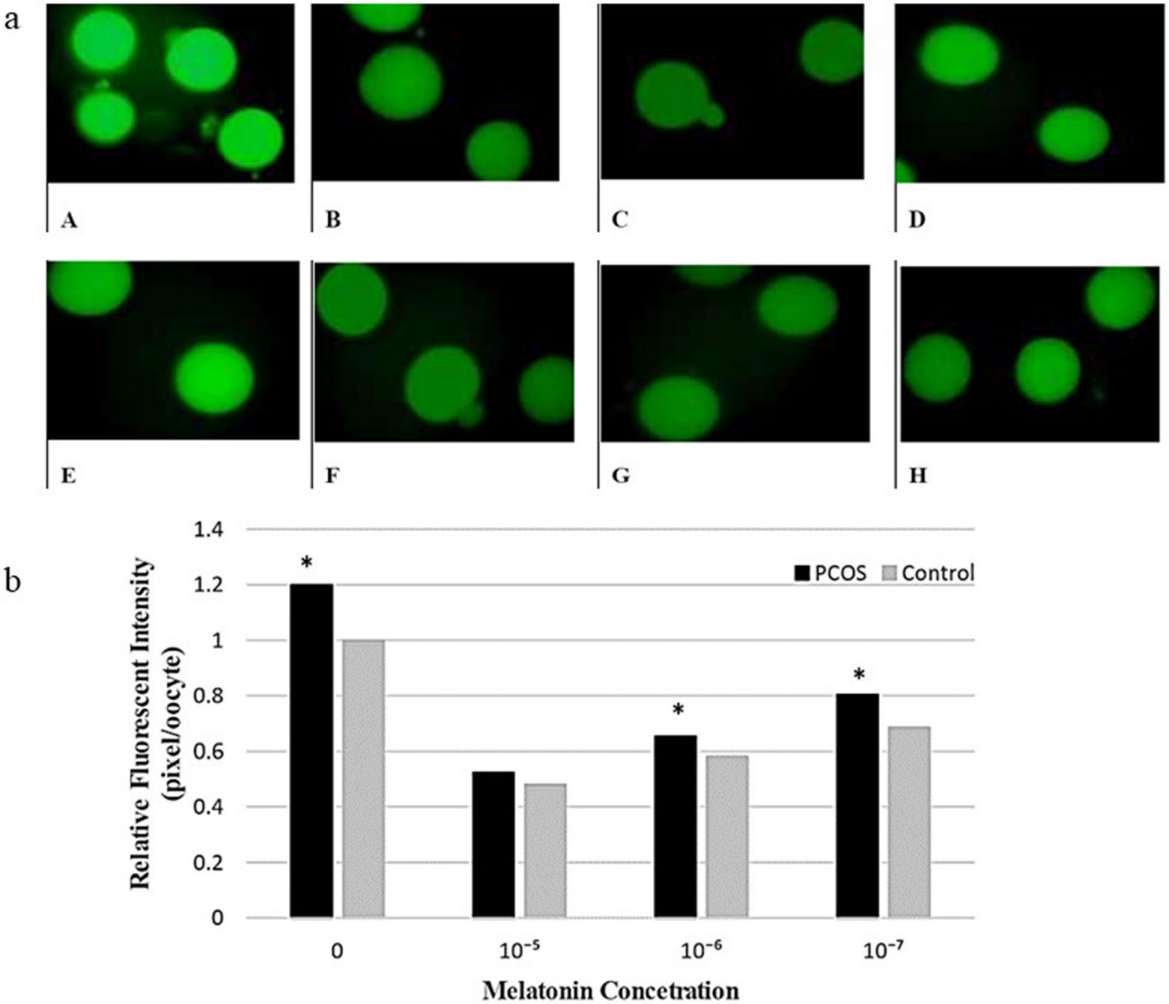
**a**: Effects of melatonin treatment on intracellular ROS levels of in-vitro matured PCOS oocytes (**A, B, C, D**) and control oocytes (**E, F, G, H**). Melatonin concentration, **A, E** (0), **B, F** (10^− 5^ M), **C, G** (10^− 6^ M), **D, H** (10^− 7^ M). **b**: The relative ROS levels in PCOS and Control groups in different melatonin concentrations

The comparison between-groups showed that the number of free radicals is significantly higher in PCOS group (*p* ≤ 0.05), regardless of the presence or absence of melatonin. The changes in the concentration of free radicals are illustrated in Fig. 3a. The intensity of the entire picture’s color was reduced to remove the background color, avoiding miscalculation of fluorescent intensity after imaging. The fluorescent intensity is shown in Fig. 3b.

## Discussion

To optimize IVM conditions, understanding molecular mechanisms that regulate oocyte maturation is fundamental. Oocyte development potential is one of the critical factors for successful fertilization, especially during the final stages of oogenesis and follicular development, during which oocyte is more susceptible to unfavorable conditions; for instance, one produces ROS and OS in PCOS. OS induces DNA damages to oocytes and its inappropriate gene expression with consequences for fertilization and embryo development [13]. Here the impact of melatonin supplementation in IVM media on expressing the following parameters: oocyte maturation-related genes (*Gdf9* and *Bmp15*), antioxidant-related genes (*Gpx1* and *Sod1*), apoptotic biomarkers (*Bcl2* and *Bax*) and intracellular total reactive oxygen species (ROS) levels, are discussed separately.

### Effect of melatonin on the expression of genes related to the oocyte development capability

The development potential of the oocyte is achieved through communication signals between oocytes and their surrounding cells, i.e., cumulus cells. Oocyte secretes oocyte soluble factors (OSF), including *Gdf9* and BMP15, that play a vital role in the cellular processes, including proliferation, differentiation, apoptosis, cumulus expansion, and its maturation process [14]. Due to the presence of melatonin receptors on the oocytes, the direct role of melatonin on oocyte has been confirmed [15].

The results of the current study showed that melatonin increases *Bmp15* expression in oocytes. There are few studies on the impact of melatonin on the expression of *Bmp15* and *Gdf9*. Consistent with our findings, Tian et al. (2014) found that the addition of melatonin during bovine oocyte maturation significantly increases *Gdf9* expression [16], in contrast, a further study on porcine oocytes showed that *Bmp15* expression was significantly elevated, not *Gdf9* [17]. Our results showed that the effect of melatonin on the expression of this gene is dose-dependent, in a way that Gdf9 is transcribed in PCOS oocytes at high concentrations of melatonin (10–5 M) and significantly decreases with the reduction of melatonin concentration. In contrast to these findings, Zhao et al. (2015) reported that melatonin reduces mRNA expression levels of Gdf9 and Bmp15 in bovine oocytes [18]. This contradiction could be due to differences in methodology because denuded oocytes from cumulus cells show low levels of Bmp15 and Gdf9 expression before maturity, whereas, in the present study, oocytes were relatively denuded before maturity. According to this study, a different expression of Bmp15 in PCOS oocytes than in the control oocytes can be due to the low quality of PCOS oocytes. Therefore, melatonin in the culture medium significantly increased Bmp15 expression. Previous studies have been shown that decreased expression of Gdf9 and Bmp15 in PCOS oocytes is known as a factor decreasing oocyte quality due to unfavorable effects of the follicular environment in PCOS [19]. Therefore, melatonin as an antioxidant increases the quality of oocytes by affecting the expression of *Bmp15* and *Gdf9*.

It is thought that high concentrations of melatonin are required to support the maturity and developmental potential of PCOS oocytes. Tamura et al. (2009) found that a decrease in melatonin level in PCOS ovaries leads to oxidative stress, which can be a factor in poor quality oocytes [20]. Our results show that melatonin can be a treatment option to protect oocytes against damages induced by follicular oxidative stress in PCOS.

### Effect of melatonin on the expression of antioxidant-related genes

The defense system, as a protective mechanism, can neutralize antioxidants or free radicals. Antioxidants are divided into enzymatic antioxidants including SOD, catalase, and GPX and non-enzymatic antioxidants such as vitamin C, taurine, hypotaurine, and glutathione [19]. Several factors regulate enzymatic antioxidants. In addition to the direct free radical scavenging activities of melatonin, it is known that melatonin is effective in the expression of antioxidant genes through suppressing or activating transcription factors on the promoter regions of antioxidant enzymes genes [21]. According to the findings of this study, in melatonin-free media, increased expression of SOD in PCOS oocytes than in the control oocytes is a stimulating factor to improve the initial defense mechanism of oocytes against ovarian free radicals. It is not the case in GPX expression pattern as found by Correa et al. (2008), that an increase in the likelihood of oxidative stress increases expression of antioxidant genes [22]. Our study clearly showed that dose-dependent melatonin significantly increases antioxidant gene expression in normal (control) and PCOS oocytes. Moreover, the expression of GPS and SOD in PCOS oocytes is mainly affected by melatonin compared to the control oocytes. However, the increase in SOD expression was not significant (Fig. 2c and d). Due to a different follicular environment in PCOS, oocytes harvested from such patients have an opportunity to compensate for increased OS by melatonin treatment. With increasing OS in the oocyte, the effect of melatonin becomes more evident, which means that melatonin may respond to a certain level of free radicals in the oocytes. The more this level increases, the more the effect of melatonin is stimulated.

According to our previous study, increased expression of antioxidant genes and reduced OS is associated with increased oocyte competency and embryo development [12]. It can be confirmed with an increase in TGFβ family gene expression that is confirmed by current results. To investigate the performance of antioxidant genes expressed in oocytes, the concentration of free radicals in oocytes was measured based on the reaction of DCFDA with H2O2. The results show that PCOS oocytes contain higher concentrations of free radicals due to improper PCOS ovarian conditions. Dose-dependent melatonin causes a reduction in free radicals in both control and PCOS oocytes. However, the free radical concentration in PCOS oocytes was higher than in the control group (Fig. 3a and b). In this context, the results of this study are consistent with those of other studies. According to Tamura (2008, 2012), melatonin is highly effective in reducing free radicals in the oocyte, and this feature depends on the dose of melatonin [20, 23]. In addition, melatonin supplementation changes the pattern of mitochondrial distribution in porcine oocytes that leads to the reduced intracellular level of ROS, all of these changes are beneficial to in vitro development of oocytes and embryo [17]. This indicates that melatonin is able to revive part of the oocyte’s ability in PCOS patients. However, at high melatonin concentrations, the amount of free radicals does not show a significant difference in PCOS and control oocytes as found for SOD expression pattern at this melatonin level. These results are consistent with other studies that have shown that reducing antioxidant activity in PCOS is due to the use of SOD antioxidants in response to the increased production of free radicals caused by increased levels of glucose and free fatty acids [24, 25].

### Effect of melatonin on the expression of apoptotic-related genes

In addition to reduced expression of antioxidant genes, the elevated OS in PCOS ovaries may be due to granulosa cells apoptosis and abnormal follicular development. On the other hand, OS has been identified as a critical factor in apoptosis. According to the result of the study by Fu; et al. (2014), melatonin in granulosa cells increases *Bcl2* expression and thereby protects these cells against apoptosis [26]. Our results also showed that melatonin results in a significant increase in the expression of Bcl2 and reduces expression of pro-apoptotic gene in the oocytes, so it has the potential ability to protect oocytes against apoptosis. High doses of melatonin in PCOS oocytes resulted in a significant increase in the expression of *Bcl2*; whereas, lower doses of melatonin did not affect expression of *Bcl2*, which may be due to the low ability of PCOS oocytes for the expression of this gene. According to the results, *Bcl2* is significantly expressed in the control oocytes as compared to the PCOS oocytes. PCOS oocytes show a different expression pattern due to the low quality PCOS oocytes. This suggests that high doses of melatonin are necessary for PCOS oocytes against apoptosis. These results are consistent with the findings of Bas et al. concerning a change in the expression of apoptotic genes, including decreased expression of *Bcl2* and increased expression of *Bax* in the PCOS model follicles [27]. Findings of this study showed a significant increase in the expression of *Bax* pro-apoptotic gene in PCOS oocytes. However, melatonin significantly decreased the expression of this gene. Dose-dependent melatonin may protect PCOS oocytes against apoptosis. It was also found that PCOS oocytes are more exposed to apoptotic damages as revealed in the expression of apoptotic genes in PCOS oocytes as compared to the control oocytes. According to Filali et al., a decrease in apoptosis in cumulus cells increases the capacity of oocytes during oocyte maturation and developmental potential [28]. Melatonin reduces apoptosis in PCOS oocytes and thereby IVM outcome is improved and this can ensure the developmental potential of oocytes. Melatonin has a dual effect on apoptosis. It has an anti-apoptotic effect in normal cells and enhances apoptosis in dying cells [29]. Results show that high concentration of melatonin affects the expression of pro- and anti-apoptotic genes and protects PCOS oocytes against apoptosis. PCOS oocytes exposed to lower concentrations of melatonin are affected by pro-apoptotic performance of melatonin due to the molecular structure and low quality of oocytes. Since the reduction of developmental potential and quality of oocytes induce apoptosis in the oocyte, melatonin may be the only factor to improve the quality of oocytes. In other words, the oocytes take the final decision to apoptosis and melatonin has no direct effect on apoptosis. Alterations in molecular mechanisms that control oocyte competence, including reduced activity of MPF and MAPK in the oocyte, decreases *Bcl2* expression and induces the onset of its apoptosis [30].

## Conclusions

According to the above findings, melatonin can generally protect PCOS oocytes against oxidative damages in IVM media, and improving oocyte competency can lead to better in-vitro embryo development. However, PCOS oocytes need more protection against oxidative damage because they positively react to high levels of melatonin in culture media. It seems that melatonin concentration in the culture media should be adjusted regarding the type and conditions of the oocytes. In this regard, medium manipulation can be a good strategy to reduce the adverse effects of follicular PCOS environment on oocytes. Further studies are necessary to investigate the molecular effects of melatonin on PCOS embryos and to examine whether oocytes influenced by melatonin can induce genomic changes in embryos or not.

## Material and methods

### Animal and treatment

The experimental protocol was approved by the ethical committee at Iran University of Medical Sciences, Tehran, Iran (NO: IUMS, 23843) and conforms to the Institutional and National Guide for the Care and Use of Laboratory Animals (National Research Council, 2011). Females pre-puberal (21–25-day-old) C57BL/6 mice were purchased from Pasteur Institute, Tehran, Iran. Animals were housed in a temperature-controlled environment in groups of two to four at 22–24 °C using an artificial light cycles (12 h light/12 h dark) and allowed free access to chow and water.

After 2 weeks of adaptation, mice were randomly divided into two groups; PCOS model group injected dehydroepiandrosterone (DHEA, s.c 6 mg/100 g bodyweight dissolved in 0.01 ml 95% ethanol and mixed with 0.09 ml olive oil), for 20 consecutive days and control group injected (s.c) 0.09 ml olive oil and 0.01 ml of 95% ethanol daily for 20 consecutive days.

Estrus cycle assessment by daily vaginal smears, hormonal assay and histological study of ovary were performed for confirming the PCOS model development as described before [12], since irregular vaginal epithelial changes in the mouse is a sign of PCOS. Briefly, vaginal cytology was assessed daily in the stable time frame (between 09.00 and 10.00 a.m.) for 4 consecutive weeks. Vaginal cells were collected and stained with Harris’ hematoxylin and eosin. Estrous stages (estrus, proestrus, metestrus and diestrus) were assessed based on vaginal cytology. Cycles with duration of 4 to 5 days were considered regular. Also, hormonal assay and histological study of ovary were performed on two mice for confirming the PCOS model induction.

### Superovulation, oocyte collection and in vitro maturation

**Fig. 4:**
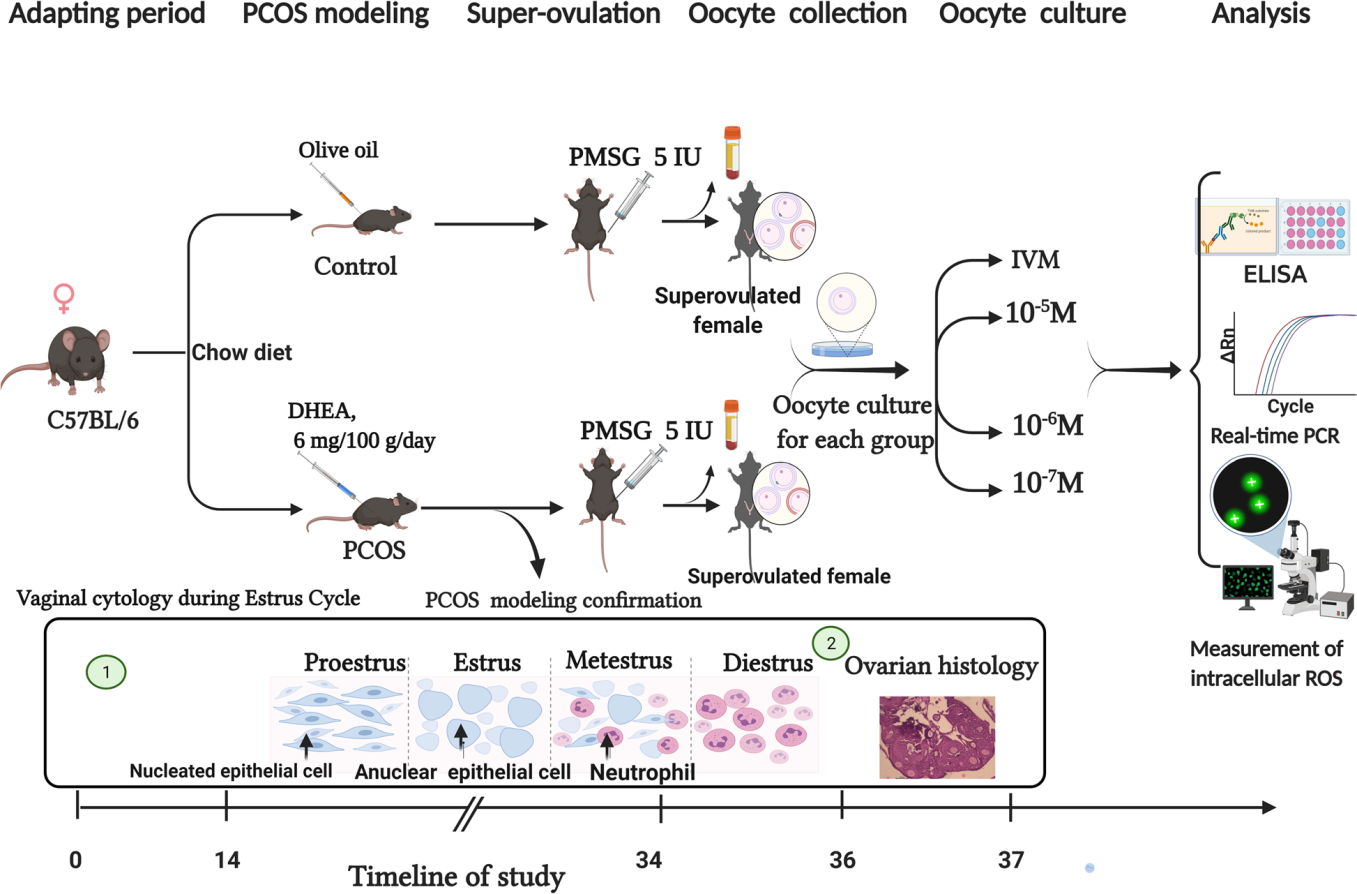
Overview of animal classification of the studied groups, modeling confirmation, and molecular analysis; created with Biorender.com

Ovarian stimulation was performed by IP injection of 5 IU pregnant mare’s serum gonadotrophin (PMSG, Sigma, St. Louis, MO, USA) to 7-week C57BL/6 female mice. Forty-eight hours after hormonal treatments, mice were euthanized by cervical dislocation, blood samples were collected into EDTA tubes by cardiac puncture, which were used for further biochemical analysis, and ovaries collected in 2.5 ml, 37° c pre-incubation handling medium (Sage/Cooper Surgical, Trumbull, CT, USA). Oocyte-cumulus were released from large antral follicles by sterile needles. After partial mechanical dissection, oocyte classified according to nuclear maturation status including germinal vesicle (GV), metaphase I and metaphase II, then GV oocytes were selected and washed three times with handling medium before transfer to IVM medium (Sage/Cooper Surgical, Trumbull, CT, USA) supplemented with a final concentration of 75 mIU FSH and 75 mIU LH at 37° c in an incubator with 5% CO2. To investigate the effects of melatonin treatment on the expression of genes related to the developmental capability of oocyte, different concentrations including 10^− 5^, 10^− 6^ and 10^− 7^ M of melatonin were added into medium culture. Approximately 10 oocytes were cultured in 1 ml IVM medium overlaid with mineral oil (Irvine Scientific, USA). After 24 h of culture, oocytes were assessed and counted for polar body (PB) extrusion (as a cellular landmark of meiotic oocyte maturation). After IVM, RNeasy Lysis Buffer (RLT buffer) was added to pools of 50–70 matured oocytes in different concentration of melatonin from both of PCOS model and control group; then they were separately snap freezed in liquid N2 before storing at − 80 °C until further analysis. Overview of animal classification to the studied groups, modeling confirmation, and molecular analysis are shown in Fig. 4.

### Hormone assay

In order to measurement of LH, FSH and testosterone serum levels, ELISA kit was used. Serum sample was collected trans-cardially at 3 h after lights on (9 a.m.), because ovulatory LH surge in mice typically occurs around the time when lights are turned off (18:30). The lowest analytical detectable levels for testosterone, FSH and LH hormones were 0.066 ng/ml, 2.81 ng/ml and 0.370 ng/ml, respectively. Intra and inter-assay coefficients of variation were < 10 and 12%, for LH, FSH and 6.5 and 11% for testosterone.

### Real-time polymerase chain reaction

The pooled oocytes were thawed (at room temperature), and centrifuged at 12000 g for 3 min in order to separate the RLT buffer. RNA extraction was performed based on the standard protocol suggested by the manufacturer (Trizol, Invitrogen, USA). The total RNA was treated with DNase I (Fermentas, Sanktleon-rot, Germany) to remove genomic DNA contamination. Complementary DNA (cDNA) was synthesized according to manufacturer’s instructions (Fermentas, Sanktleon-rot, Germany). Relative gene expression was calculated as the abundance ratio of each target gene to the housekeeping gene (β-actin). The primer pair sequences are listed in Table 1. Each PCR reaction sample consisted of 5 μL of Power SYBR Green PCR Master Mix, 11 μL dH2O, 1 μL of each of the forward and reverse primers (pmol), 2 μL of single-strand cDNA in a final reaction volume of 20 μL. The expression level of each target gene was calculated by the relative standard curve and ^ΔΔ^Ct methods.

**Table 1:** Primer sequences used for real time PCR analysis

Gene (***Mus musculus***)	Forward primer(5′-3′)	Reverse primer(5′-3′)	Annealing temperature(°C)	Product size (bp)
***Gdf9***	AAAGAAGACTGGCACGAGGA	CAAAGCAGCAAACCCCCAA	59	92
***Bmp15***	TGGGTTCCTAAATGCCGGAC	TGCCTTTTTGCTGCTGACAC	60	181
***Gpx1***	TGCAATCAGTTCGGACACCA	TCTCACCATTCACTTCGCACT	59	130
***Sod1***	AGCATTCCATCATTGGCCGT	TCCCAGCATTTCCAGTCTTTGT	60	99
***Bcl2***	AATTGTAATTCATCTGCCGCCG	GTGGAGGAAAAATCAGGAGGGT	60	150
***Bax***	ACATGGCAGACAGTGACCAT	AAGACACAGTCCAAGGCAGT	59	103
**β-actin**	AAGTACTCTGTGTGGATCGGTG	GGTGTAAAACGCAGCTCAGTAA	59	153

### Measurement of intracellular ROS

To measurement of ROS content, mature oocytes of each treatment group were transferred to a drop containing 10 μM 2′, 7′ dichlorodihydroflourescein diacetate (H2DCFDA, D6883, Sigma -Aldrich; a nonfluorescent, cell-permeable compound used as a marker of OS) for 20 min at 37 °C in the dark. After incubation, oocytes were washed in culture medium and transferred to 10 μl drop. Green fluorescence was detected for oxidase H2DCFDA using a fluorescence microscope with an UV filter (460 nm). Florescence intensity was measured using Image J software. The fluorescence emission of each oocyte was calculated and normalized to that of control group. Background fluorescent values were subtracted from the final values before analyzing for the statistical difference among the groups. Each group (PCOS and control) consists of 20–25 oocytes.

### Statistical analysis

One-way ANOVA was used to quantitative variables analysis between the two groups with different melatonin concentrations. The effect of different concentrations of melatonin on ROS levels was analyzed with Non-parametric Mann-Whitney and Kruskal-Wallis test. The level of significance was set at *P* ≤ 0.05.

## Data Availability

All data generated or analyzed during this study are included in the manuscript.

## Abbreviations

PCOS: Polycystic ovary syndrome
ART: Assisted reproductive techniques
IVM: In-vitro maturation
OS: Oxidative stress
ROS: Reactive oxygen species
DHEA: Dehydroepiandrosterone
SOD: Superoxide dismutase
